# Mass spectrometry–driven exploration reveals nuances of neoepitope-driven tumor rejection

**DOI:** 10.1172/jci.insight.129152

**Published:** 2019-07-25

**Authors:** Hakimeh Ebrahimi-Nik, Justine Michaux, William L. Corwin, Grant L.J. Keller, Tatiana Shcheglova, HuiSong Pak, George Coukos, Brian M. Baker, Ion I. Mandoiu, Michal Bassani-Sternberg, Pramod K. Srivastava

**Affiliations:** 1 Department of Immunology and Carole and Ray Neag Comprehensive Cancer Center, University of Connecticut School of Medicine, Farmington, Connecticut, USA.; 2 University of Lausanne, Lausanne, Switzerland.; 3 Department of Oncology, University Hospital of Lausanne, Lausanne, Switzerland.; 4 Department of Chemistry and Biochemistry and Harper Cancer Research Institute, University of Notre Dame, Notre Dame, Indiana, USA.; 5 Department of Computer Sciences, University of Connecticut School of Engineering, Storrs, Connecticut, USA.

**Keywords:** Immunology, Antigen presentation, Dendritic cells, Immunotherapy

## Abstract

Neoepitopes are the only truly tumor-specific antigens. Although potential neoepitopes can be readily identified using genomics, the neoepitopes that mediate tumor rejection constitute a small minority, and there is little consensus on how to identify them. Here, for the first time to our knowledge, we use a combination of genomics, unbiased discovery mass spectrometry (MS) immunopeptidomics, and targeted MS to directly identify neoepitopes that elicit actual tumor rejection in mice. We report that MS-identified neoepitopes are an astonishingly rich source of tumor rejection-mediating neoepitopes (TRMNs). MS has also demonstrated unambiguously the presentation by MHC I, of confirmed tumor rejection neoepitopes that bind weakly to MHC I; this was done using DCs exogenously loaded with long peptides containing the weakly binding neoepitopes. Such weakly MHC I–binding neoepitopes are routinely excluded from analysis, and our demonstration of their presentation, and their activity in tumor rejection, reveals a broader universe of tumor-rejection neoepitopes than presently imagined. Modeling studies show that a mutation in the active neoepitope alters its conformation such that its T cell receptor–facing surface is substantially altered, increasing its exposed hydrophobicity. No such changes are observed in the inactive neoepitope. These results broaden our understanding of antigen presentation and help prioritize neoepitopes for personalized cancer immunotherapy.

## Abstract 2

Neoepitopes identified by mass spectrometry are a rich source of tumor rejection antigens, including those with a weak binding to MHC I.

## Introduction

The ability of tumors to elicit potent tumor-rejecting immune response in syngeneic mice was demonstrated over 70 years ago (1–3). These studies contained the additional and then strange observation that each individual tumor was antigenically distinct. The individuality of tumors was confirmed in several later studies, often in a dramatic fashion (4, 5), suggesting the possibility that each new tumor that arose harbored a unique antigen. To provide a possible mechanistic basis for the existence of an apparently limitless universe of individual tumor rejection antigens, it was suggested more than 25 years ago that individual tumor-specific antigens are MHC I–presented mutant peptides (neoepitopes) that derive from random passenger mutations in cancer cells (6). The earliest neoepitopes of mouse and human cancers were defined using T cells as probes, and an overwhelming majority of these neoepitopes did turn out to be mutant peptides (7–12). Recent genomic and bioinformatic studies have confirmed that neoepitopes derived from random passenger mutations in cancer cells can indeed serve as tumor rejection antigens (13–16). These studies have also shown clearly that only a very small proportion of the many nonsynonymous mutations (mutations that result in change in an amino acid) in a tumor actually encode a tumor rejection–mediating antigen.

The rules that may be used to identify the actual tumor rejection–mediating neoepitopes (TRMNs) from the vast majority of non-TRMNs (n-TRMNs) that result from the many mutations are unclear. The predicted binding affinity of the mutant neoepitope peptides for the MHC alleles of the tumor cells is often used as the single most important parameter to distinguish the true TRMN from the n-TRMN (14–16). However, the utility of affinity for that purpose has been disputed in mouse (13) as well as human studies (17, 18). Bioinformatic data alone cannot predict which of the many possible candidate neoepitopes will actually be presented by MHC proteins. Indeed, Assarsson et al. (19) showed that the proportion of peptides actually presented among all possible peptides may be on the order of 15%.

Recently, mass spectrometry (MS) has been suggested as an alternative tool that can help identify TRMNs. MS is the only tool that can identify the peptides that are naturally processed and presented by MHC molecules, albeit with limitations (20). Yadav et al. (14) demonstrated the utility of MS in enriching TRMNs among a pool of candidate neoepitopes of a mouse carcinoma, and Gubin et al. applied targeted MS analysis to confirm presentation of predicted yet confirmed immunogenic neoepitopes in a mouse sarcoma (15). Using clinical samples of human melanoma, Bassani-Sternberg et al. (21) identified nearly 100,000 peptides presented by the HLA molecules; of those, 11 were shown to be neoepitopes, and of the 11, 4 elicited CD8^+^ T cell responses in the patient.

Mouse models allow deep mechanistic exploration through testing the ability of large numbers of peptides for their ability to elicit actual tumor rejection in addition to immune responses, and to use this information to divine the rules of anti-tumor immunity. In humans, such information can only be acquired through long clinical trials. Here, for the first time, we use a combination of genomics, unbiased discovery MS immunopeptidomics, and targeted MS with labeled standards for validation to directly identify neoepitopes that result in actual tumor rejection and we further demonstrate the powerful potential of MS as a screening tool. MS analysis shows here for the first time to our knowledge that peptides with extremely low affinity for MHC I proteins (IC_50_ as low as 10,000 to 40,000 nM) can be presented by MHC I proteins. In addition, we demonstrate the advantages provided by structural modeling, which provide further insight into how various mutations can act to yield potent epitopes. Our studies reveal unexpected nuances in our understanding of characteristics of TRMNs, including observations that peptides that would traditionally be excluded based on predicted MHC binding and sequence characteristics can indeed be highly relevant. These nuances should be taken into consideration when prioritizing targets for personalized neoepitope-based cancer vaccines and cancer immunotherapy.

## Results

### Direct identification of neoepitopes by MS-based immunopeptidomics.

MS-based immunopeptidomics analysis was performed on MHC class I (MHC I) complexes eluted from the BALB/cJ fibrosarcoma Meth A cells to directly identify naturally presented neoepitopes. We searched the resulting tandem MS (MS/MS) data of the eluted peptides against a reference database of the mouse proteome concatenated to a list of 3,783 long peptides encompassing nonsynonymous somatic mutations predicted for this tumor by our prediction pipeline Consensus Caller Cross Platform (CCCP). With the data-dependent acquisition method, we identified a total of 6,209 MHC I–bound peptides applying an FDR of 5% for peptide identification (Supplemental Table 1; supplemental material available online with this article; https://doi.org/10.1172/jci.insight.129152DS1). The peptides fit the expected peptide length distribution as well as the MHC-binding specificities; representative results are shown for the K^d^, D^d^, and L^d^ MHC I molecules (Supplemental Figure 1, A and B). Of the MHC I–eluted peptides identified by MS/MS, 8 were determined to be neoepitopes, with binding restriction to K^d^ or D^d^ MHC I molecules, as determined by the binding specificities of these alleles (Supplemental Figure 1B). These were independently identified in several immunopeptidomics experiments. The sequences of these neoepitopes, along with their non-mutated counterparts, the predicted MHC I–binding affinities for cognate alleles, their Differential Agretopic Indices (DAI; ref. 13), and their RNA expression are shown in Table 1. The MS intensity of the eluted neoepitopes was relatively similar to the intensity of the non-mutated peptides (Supplemental Figure 1C).

### Validation of the detected neoepitopes.

**Figure 1:**
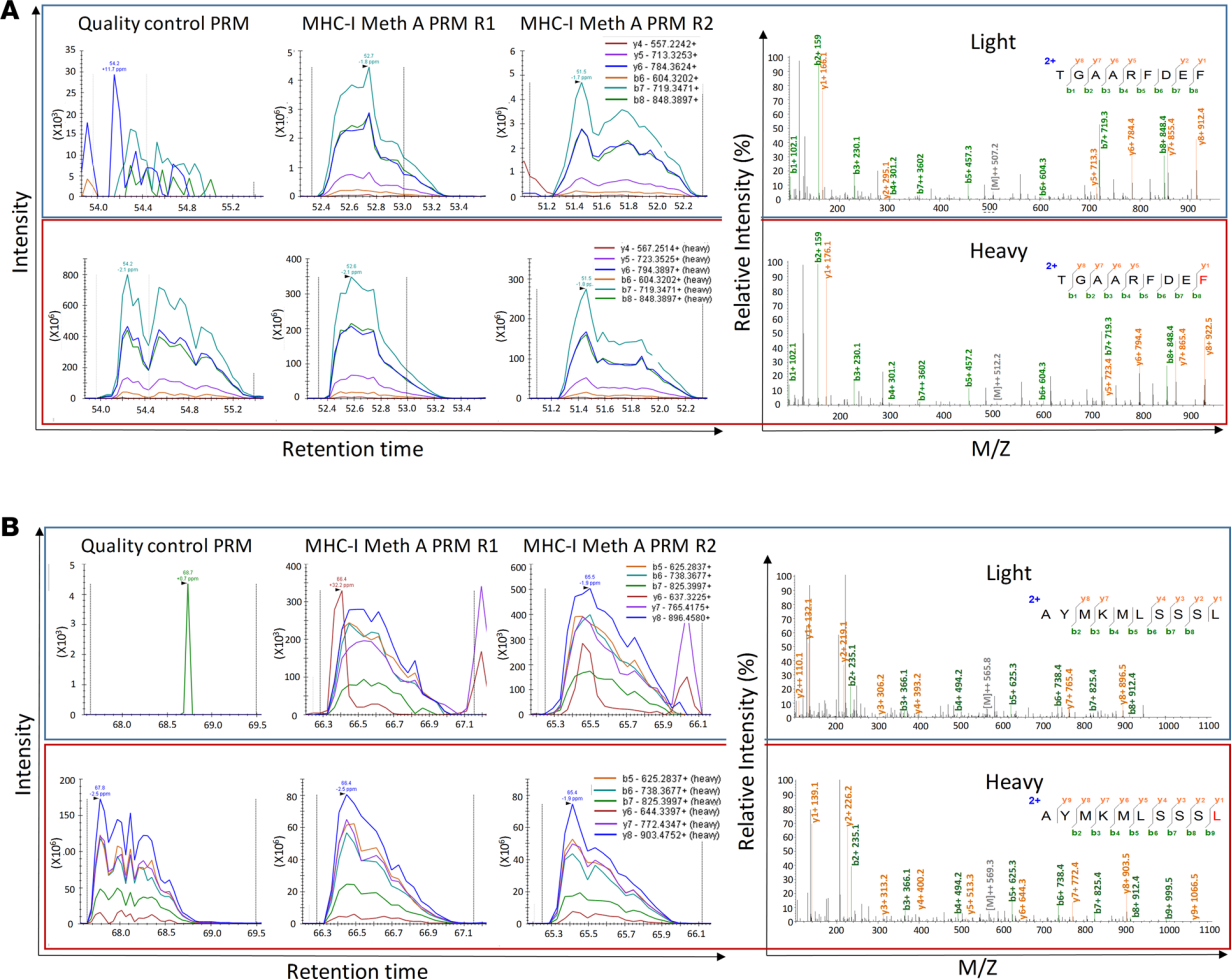
Targeted MS–based validation of the MS-identified MHC I neoepitopes TGAARFDEF and AYMKMLSSSL. (**A**) TGAARFDEF. (**B**) AYMKMLSSSL. Matched peak lists for the “heavy” and “light” ions were extracted and monitored. First, the absence of “light” peptide and the presence of the “heavy” peptide were confirmed by PRM as a quality control measure in the synthetic peptide samples (upper left and lower left, respectively). Then, the co-elution of the synthetic “heavy” and endogenous “light” fragment ions was measured by PRM in 2 independent Meth A MHC I immunopeptidomics samples. Representative resulting MS/MS spectra of the “light” and “heavy” counterparts are provided. Figures were edited to improve resolution and readability.

For validation of the detected neoepitopes, their corresponding mutations were confirmed by Sanger sequencing of Meth A genomic DNA. In addition, we spiked synthetic peptide counterparts that were stably labeled with heavy isotopes (“heavy” peptides) into additional samples of MHC I peptides eluted from the Meth A cells (“light” peptides). We applied the parallel reaction monitoring (PRM) acquisition method to target with high sensitivity and accuracy, co-eluting pairs of heavy and light peptides. The data for confirmation of 2 neoepitopes, TGAARFDEF (derived from *Gtf2b*) and AYMKMLSSSL (derived from 1190007I07Rik), are shown in Figure 1; the data for the remaining 6 neoepitopes are shown in Supplemental Figure 2. We thus confirmed the identification of the 8 MHC I neoepitopes. As a negative control, we performed a similar immunopeptidomics approach on BALB/cJ antigen-presenting cells (APCs), resulting in the identification of 1,166 MHC I–binding peptides (5% FDR). None of the neoepitopes were detected in the discovery or PRM MS measurements.

### Tumor rejection activity of MS-defined neoepitopes.

**Figure 2:**
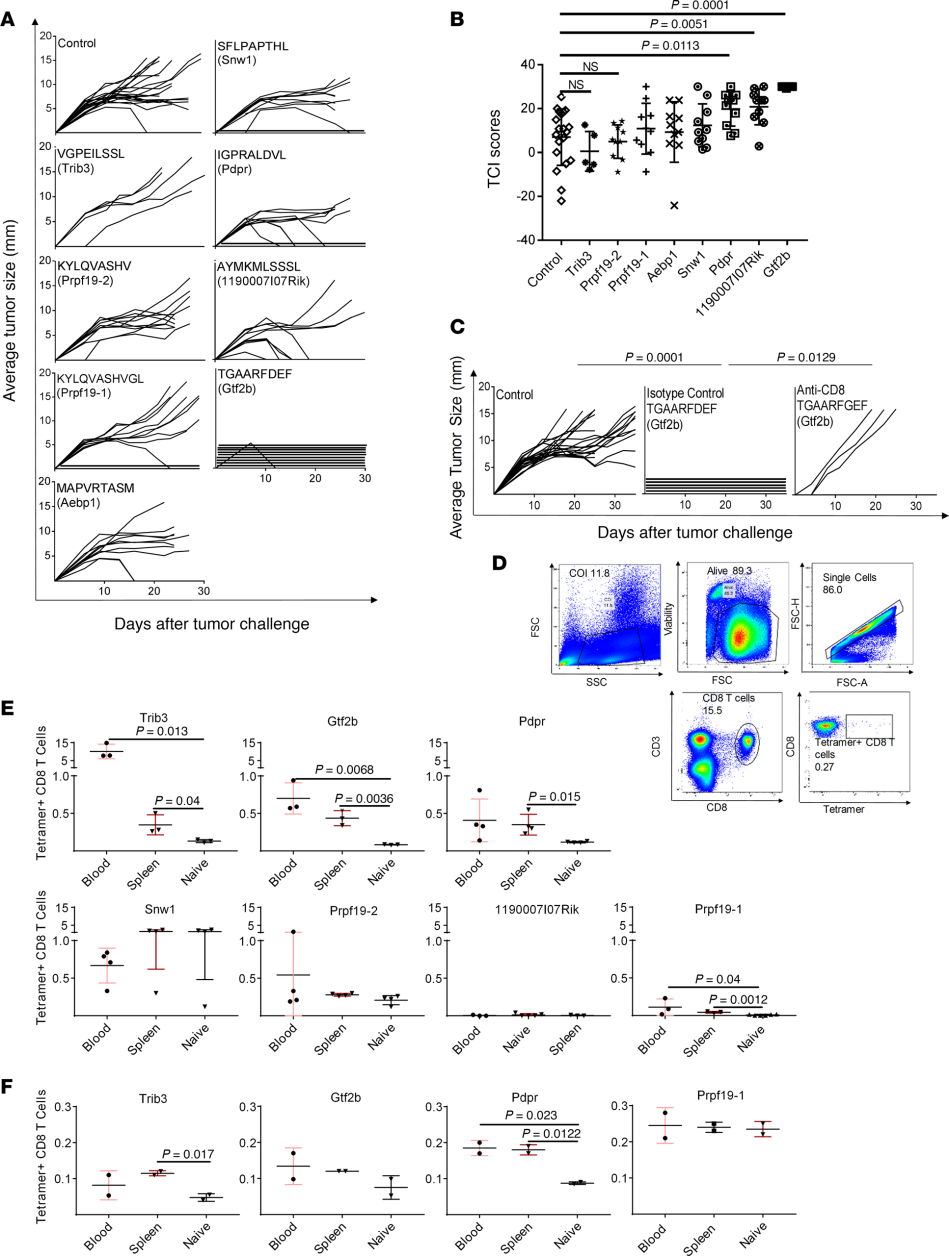
Tumor rejection and CD8^+^ tetramers elicited by immunization with neoepitopes. (**A**) Naive BALB/cJ mice were immunized, as described in the Methods, with the 8 neoepitopes identified in Table 1, and were challenged with 95,000 Meth A cells. Tumor growth was measured. Each line represents tumor growth in a single mouse. (**B**) Total TCI scores for data in **A**. The maximum possible TCI score is 30. Each column shows the average total TCI score for the indicated treatment group. Error bars are shown above each column. *P* values for TCI scores comparisons were calculated using 1-way ANOVA test. Dunnett’s test was performed as a follow-up test to ANOVA to correct for multiple comparisons. The adjusted *P* values are reported. The experiment was repeated 3 or more times. (**C**) BALB/cJ mice were injected with CD8-depleting antibody (or isotype control) as described in the Methods. Immunization (with the indicated neoepitope) and tumor challenge were as in **A**. *P* values for comparison of the AUC scores were calculated using 1-way ANOVA. Tukey’s test was performed as follow-up test to ANOVA to correct for multiple comparisons. The adjusted *P* values are reported. (**D**) Gating strategy for splenocytes and blood cells of immunized mice stained for CD3, CD8, and tetramer. (**E**) Naive BALB/cJ mice were immunized with each of 7 indicated neoepitopes, and blood and spleen of individual mice were tested for presence of tetramer^+^ CD8^+^ T cells. In most instances, the responses shown for naive mice are the averages of the responses in blood and spleen, which were very similar to each other. *P* values for CD8^+^ T cell response comparisons were calculated using a 2-tailed *t* test. *P* < 0.05 was considered statistically significant. (**F**) Blood and spleen of individual non-immunized tumor-bearing BALB/cJ mice were tested for the presence of tetramer^+^ CD8^+^ T cells for the 4 neoepitopes in **E**, which elicited tetramer^+^ CD8^+^ T cells in immunized tumor-naive mice. *P* values for CD8^+^ T cell response comparisons were calculated using a 2-tailed *t* test. The experiment was repeated 3 or more times.

The 8 MS-defined neoepitopes were used to immunize naive BALB/cJ mice twice, 1 week apart, with neoepitope-pulsed bone marrow–derived dendritic cells (BMDCs, 3 × 10^6^) as adjuvants, as previously published (22). All mice were challenged with 95,000 Meth A tumor cells 1 week after the second immunization, and tumor growth was monitored in individual mice (Figure 2, A and B). Two neoepitopes, TGAARFDEF and AYMKMLSSSL, showed the highest tumor rejection capacities among the 8 mutant peptides tested. Mice immunized with TGAARFDEF showed complete tumor rejection (*P* < 0.0001). Among mice immunized with AYMKMLSSSL, 60% of the immunized mice were able to reject the tumor completely, and another 30% showed relatively prolonged tumor stabilization (*P* = 0.0051). Another neoepitope, IGPRAVDVL (derived from *Pdpr*), showed statistically significant tumor control and an intermediate tumor rejection activity (*P* = 0.113). Figure 2B shows the tumor control indices (TCI; 23) of the data in Figure 2A, and their statistical significance. The TCI score parameterizes the tumor rejection, tumor inhibition, and tumor stability scores to produce a total TCI score, which reflects the control of the tumor growth in each group.

CD8 dependence of neoepitope-elicited tumor immunity was tested in the case of the peptide TGAARFDEF (derived from *Gtf2b*) by depletion of neoepitope-immunized mice by anti-CD8 depleting antibody or an isotype control antibody during the priming phase alone, as described in the Methods. Depletion of CD8^+^ T cells completely abolished the neoepitope-elicited tumor rejection in 3 of the 5 mice tested (Figure 2C). The kinetics of recovery of the depleted CD8^+^ cells in mice is not uniform, resulting in lack of abrogation of immunity in 2 of the 5 mice. Because depletion of CD4^+^ T cells results in depletion of helper as well as regulatory CD4^+^ T cells, and the 2 effects are contradictory, depletion of CD4^+^ cells was not attempted.

In parallel, naive, non–tumor-bearing mice immunized with the neoepitopes were tested for CD8^+^ T cell responses to 7 of the 8 neoepitopes. ELISpot assays could not be performed because of the high background generated by incubation of T cells with any DCs. CD8^+^ cells from mice immunized with individual peptides were tested for the presence of IFN-γ^+^ cells; a specific response was detected in mice immunized with TGAARFDEF (from *Gtf2b*) but not with any other neoepitopes (data not shown). Tetramer staining was therefore used for better sensitivity and specificity (Figure 2D). (Tetramers against the neoepitope MAPVRTASM derived from *Aebp1* gene could not be made.) Blood and the spleen of the immunized mice were harvested 1 week after the second immunization and stained for CD3, CD8, as well as specific binding to neoepitope tetramers (Figure 2, D and E). Of the 2 neoepitopes able to strongly elicit tumor rejection (TGAARFDEF and AYMKMLSSSL), only TGAARFDEF elicited a measurable CD8^+^ T cell response as detected by tetramer staining. In the blood and spleen of TGAARFDEF-immunized mice, tetramer^+^ cells comprised approximately 0.7% and 0.43% of the total CD8^+^ T cells, respectively; these numbers are significantly higher than the corresponding numbers in non-immunized mice (*P* = 0.0068 and 0.0036 in the blood and spleen, respectively). The neoepitope AYMKMLSSSL, which elicited complete tumor rejection in 60% of the immunized mice, did not elicit a measurable CD8^+^ response. The intermediate neoepitope IGPRAVDVL elicited measurable and significant CD8^+^ T cell response (~0.35% tetramer^+^ cells, *P* = 0.015). Of the 5 MHC I neoepitopes that did not elicit tumor rejection, 2 elicited a measurable CD8^+^ T cell response. Of these, the response elicited by VGPEILSSL (derived from *Trib3*) was the strongest CD8^+^ T cell response among all neoepitopes; approximately 10% of the CD8^+^ T cells (in blood) in mice immunized with it were tetramer^+^. As controls, we measured, in 2 instances, tetramer response in mice immunized with an irrelevant neoepitope; such nonspecific response was never detected.

Thus, of the 7 neoepitopes tested, 4 neoepitopes elicited expansion of tetramer^+^ CD8^+^ T cells on immunization of naive, non–tumor-bearing mice (Figure 2E). We now examined CD8^+^ T cell responses to the 4 neoepitopes in unimmunized, tumor-bearing mice (Figure 2F). Surprisingly, we could not detect a measurable CD8^+^ T cell response in tumor-bearing mice against TGAARFDEF, the neoepitope that elicited the strongest tumor rejection (Figure 2A) and a robust CD8^+^ T cell response in non–tumor–bearing immunized mice (Figure 2E). The neoepitope VGPEILSSL (which elicited the strongest CD8^+^ T cell response but no tumor rejection on immunization of naive mice in Figure 2, A and E) showed significant CD8^+^ T cell response in tumor-bearing mice (Figure 2F). The neoepitope IGPRALDVL, which elicited intermediate tumor rejection (Figure 2A) and a good tetramer CD8^+^ T cell response in naive mice (Figure 2F), elicited significant CD8^+^ T cell response in tumor-bearing mice as well (Figure 2F). Finally, the neoepitope KYLQVASHVGL, which did not elicit tumor rejection but elicited a strong CD8^+^ T cell response in naive mice, did not elicit such a response in tumor-bearing mice. As controls, CD8 cells from mice bearing an antigenically irrelevant tumor (4T1) were tested for tetramer response to all 7 neoepitopes; no response was detected. Altogether, CD8^+^ T cell responses in non-immunized tumor-bearing mice and immunized tumor-naive mice were often discordant.

### MS analysis of low-abundance TRMNs.

**Figure 3:**
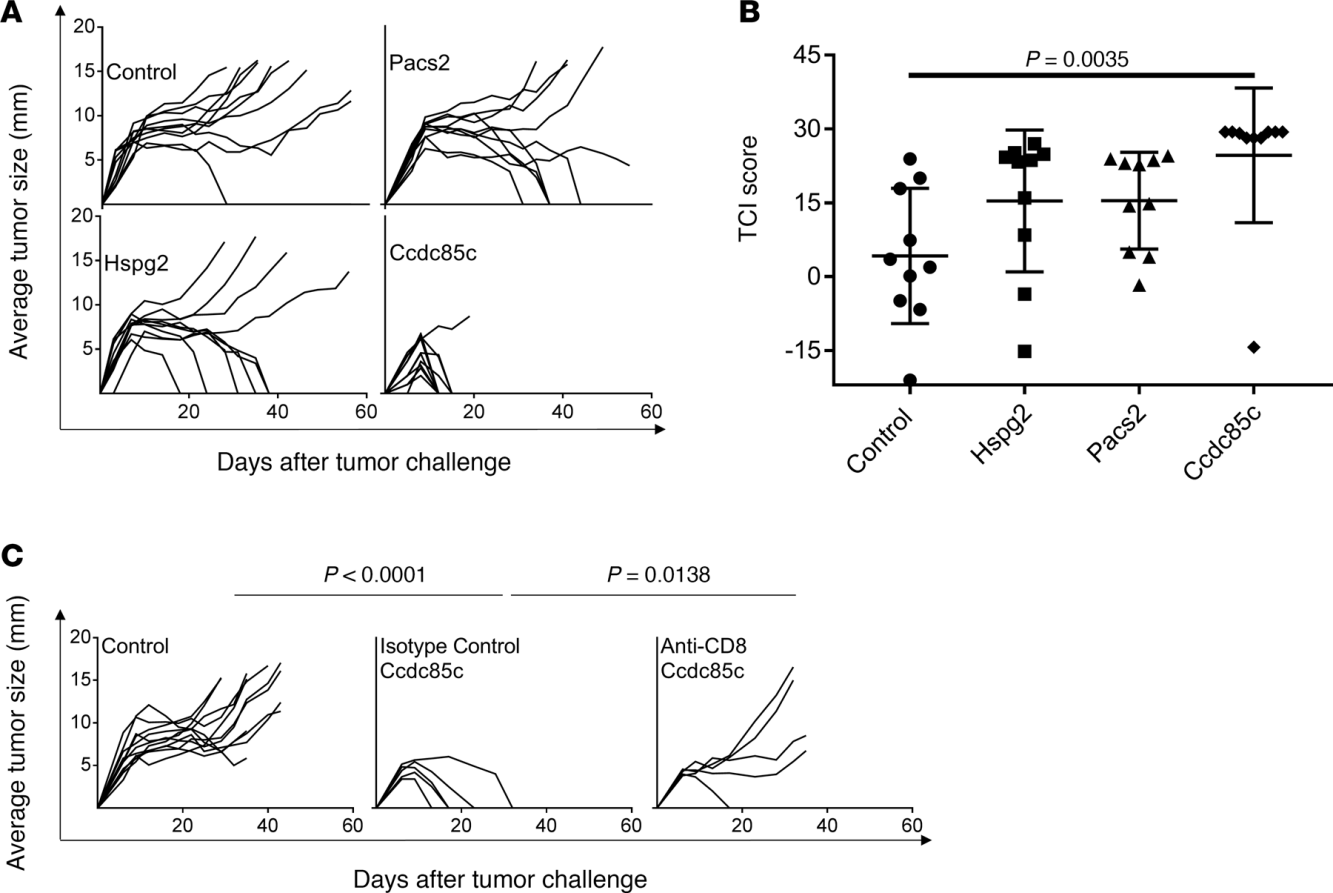
Tumor rejection elicited by immunization with low-abundance TRMNs. (**A**) Naive BALB/cJ mice were immunized, as described in Methods, with the 3 neoepitopes identified in Table 2, and were challenged with 95,000 Meth A cells. Tumor growth was measured. Each line represents tumor growth in a single mouse. (**B**) Total TCI scores for data in **A**. Each column shows the average total TCI score for the indicated treatment group. Error bars are shown above each column. *P* values for TCI scores comparisons were calculated using 1-way ANOVA test. Dunnett’s test was performed as a follow-up test to ANOVA to correct for multiple comparisons. The adjusted *P* values are reported. The experiment was repeated 3 or more times. (**C**) BALB/cJ mice were injected with CD8-depleting antibody (or isotype control) as described in Methods. Immunization (with indicated neoepitope) and tumor challenge were carried out as in **A**. *P* values for comparison of the area under the curve (AUC) scores were calculated using 1-way ANOVA. Tukey’s test was performed as a follow-up test to ANOVA to correct for multiple comparisons. The adjusted *P* values are reported.

In parallel with the use of MS to identify TRMNs, we have tested a series of other candidate neoepitopes (without help from MS) for tumor rejection activity. In these studies, we identified 2 additional TRMNs from this same tumor by applying prediction tools (13), without the aid of MS (Table 2). Two neoepitopes, TYIRPFETKVK (derived from *Ccdc85c*) and HYLSSILRL (derived from *Pacs2*) showed the highest tumor rejection capacities (Figure 3). Mice immunized with TYIRPFETKVK showed almost complete tumor rejection (*P* = 0.0035). Among mice immunized with HYLSSILRL or SYLLGSGEARL (derived from *Hspg2*), about 50% of the immunized mice were able to reject the tumor completely but the activity was not significant because of a high variation in tumor rejection. Immunity elicited by the TYIRPFETKVK (from *Ccdc85c*) was observed to be CD8 dependent by depletion assay in vivo (Figure 3C).

Based on the abundance of RNA encoding the proteins harboring these TRMNs, these TRMNs do not appear to be generally abundant. Indeed, these neoepitopes could not be identified among the Meth A MHC I–eluted peptides identified by MS, presumably consistent with the sensitivity bias of the MS approach. All but 2 of these TRMNs have low predicted and measured affinity for MHC I molecules, ranging in IC_50_ values between 1,433 nM and 39,661 nM (Table 2). We used MS to determine whether these low-affinity TRMNs could be processed and presented by MHC I. Long peptides containing the TRMNs were pulsed on BMDCs and excess peptides were washed off. Peptides were eluted from the MHC I molecules purified from the pulsed DCs and were analyzed by MS. We detected a rather limited repertoire of cross-presented MHC I ligands. Importantly, multiple precise TRMNs, which had been demonstrated experimentally to mediate tumor rejection (Supplemental Table 2), were able to be eluted from the MHC I of the pulsed BMDCs (Table 2). These neoepitopes reside in genes with low-abundance transcripts (Table 2) and could not be detected by MS among naturally presented peptides; they could only be shown to be presented when loaded with high amounts of synthetic peptide on DCs. These results (a) demonstrate the definitive identity of the precise neoepitope in these TRMNs, and (b) prove unequivocally that on loading, these TRMNs with low affinity for MHC I are nonetheless presented by MHC I molecules.

### Molecular modeling of an active and an inactive neoepitope.

The activity of TRMNs will in part be influenced by features that distinguish them from their WT counterparts and drive recognition by αβ T cell receptors. To provide insight into any such features, we modeled the structures of the most active (TGAARFDEF) neoepitope and an inactive (KYLQVASHV) neoepitope and their WT counterparts bound to the presenting MHC I.

**Figure 4:**
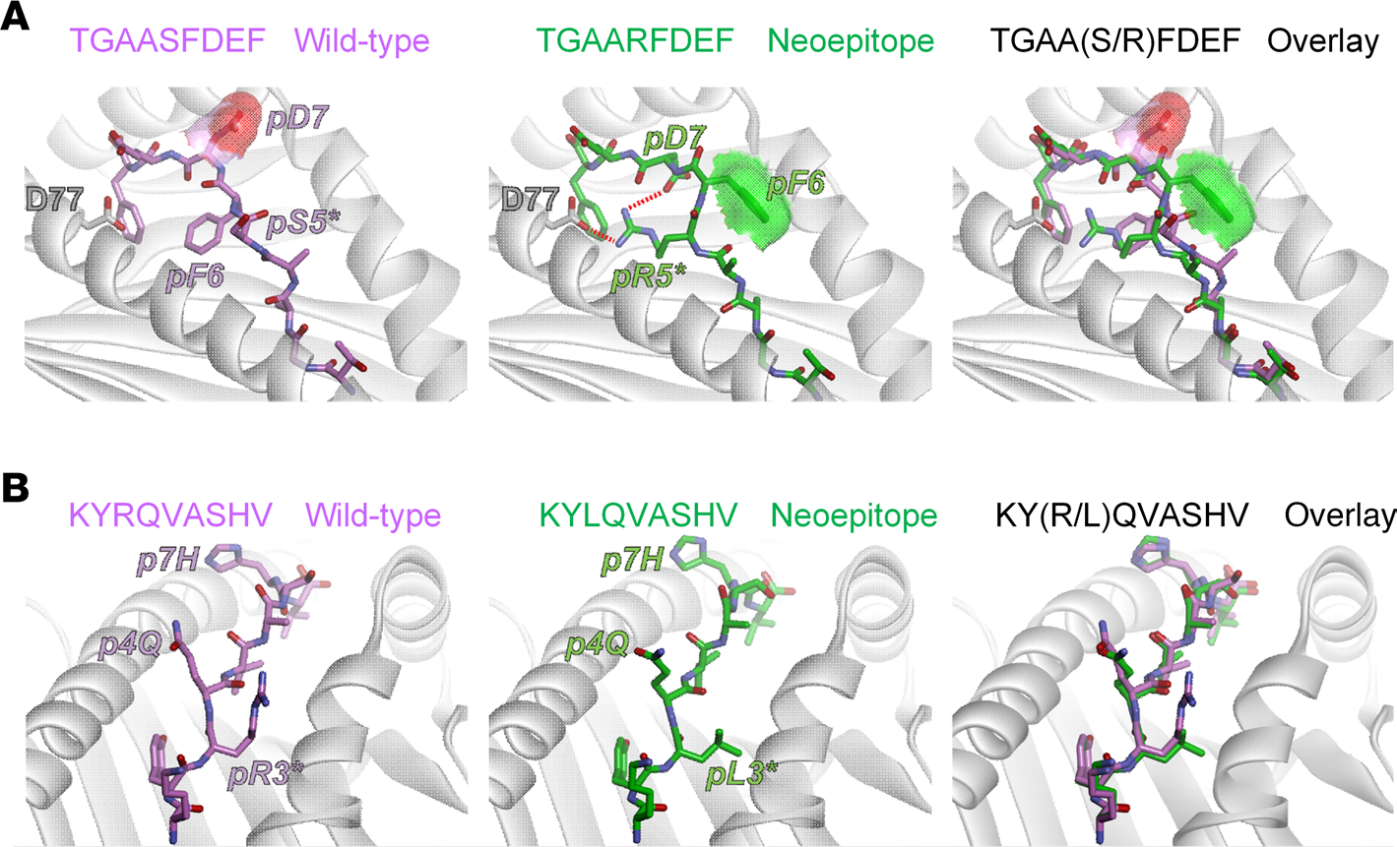
Models of peptide/MHC complexes indicate structural and physical correlates with immunogenicity. (**A**) For the tumor rejecting TGAARFDEF neoepitope, the serine-to-arginine substitution at position 5 is predicted to alter peptide conformation and increase exposed hydrophobic surface area. In the model of the wild-type complex (left panel), Phe6 acts as a secondary anchor. Asp7 is solvent exposed, as indicated by the red surface. In the neoepitope complex (middle panel), the new arginine at p5 takes on the secondary anchor role, forming a salt-bridge with Asp77 of the H2-D^d^ α1 helix. The switch in secondary anchor from p6 to p5 results in Asp7 shifting down toward the base of the groove to form a second salt-bridge with Arg5, reducing its solvent exposure. Coincident with this shift, Phe6 moves up to become solvent exposed, as indicated by the green surface. An overlay of the neoepitope and its wild-type counterpart demonstrates the substantial differences between the two (right panel). (**B**) For the inactive KYLQVASHV neoepitope, the arginine-to-leucine substitution at position 3 does not alter peptide conformation. In the model of the wild-type complex (left panel), Arg3 is positioned between the peptide backbone and the H2-K^d^ α2 helix. Gln4 and His7 are exposed and face up toward incoming TCRs. In the neoepitope complex, the new leucine at p3 simply fills the same space between the peptide and the α2 helix and does not introduce any structural alterations to the peptide (middle panel). An overlay of the neoepitope and its wild-type counterpart demonstrates the similarities between the wild-type peptide and neoepitope.

The TRMN TGAARFDEF incorporates a serine-to-arginine mutation at P5, which is typically a secondary anchor in MHC I proteins. Phenylalanine is preferred for strong D^d^-binding peptides, although studies have indicated that arginine, isoleucine, and glycine are also tolerated (24). Indeed, in the model of the TGAARFDEF/D^d^ complex, the mutant arginine serves as a secondary anchor, forming a salt bridge with Asp77 of the D^d^ α1 helix (Figure 4A). In the model with the WT peptide, however, this role is played by the phenylalanine at P6. The switch between the use of P6 (WT) versus P5 (neoepitope) as an anchor results in a large change in the conformation of the peptide backbone from P4 to P7 (the WT and neoepitope peptide backbones superimpose with a root mean square (RMS) deviation of 1.3 Å; for all common atoms, the value is 2.8 Å). The strain relieved by this switch can help explain the improved MHC binding of the TGAARFDEF neoepitope compared with the WT peptide. Most interestingly though, the change in peptide conformation results in exposure of the P5 phenylalanine, whereas the P7 aspartic acid becomes buried at the base of the groove where it also interacts with the new P5 arginine. The net result is that, in addition to enhancing peptide binding affinity, the serine-to-arginine mutation in TGAARFDEF increases the exposed hydrophobic surface area of the peptide by 25% (from 111 Å^2^ to 139 Å^2^). Notably, the exposed hydrophobic surface is associated with T cell receptor recognition of antigenic epitopes (25, 26).

The inactive KYLQVASHV neoepitope incorporates an arginine-to-leucine mutation at P3. In MHC I complexes, the P3 side chain is typically packed against the α2 helix, as is the case in the models for both the neoepitope and its WT counterpart (Figure 4B). In the models, the leucine adopts the same conformation as the first half of the WT arginine side chain, but with its branched side chain packs more tightly against the helix. This tighter packing can explain the slightly improved K^d^ binding affinity of the neoepitope compared with the WT. The mutation, however, is not predicted to alter the conformation of the peptide in any fashion (backbone RMS deviation of 0.4 Å). In addition, for both the neoepitope and WT peptides, the polar side chains at P4 (glutamine) and P7 (histidine) are predicted to face upwards toward incoming T cell receptors in approximately the same orientation, which might explain the lack of anti-tumor activity of the KYLQVASHV neoepitope despite the improved peptide-MHC binding affinity.

## Discussion

Identification of neoepitopes that can stimulate host immune response to cancer leading to modulation of the course of tumor growth is the holy grail of cancer immunotherapy. To the extent that these neoepitopes are unique to individual cancers makes the search for this holy grail that much more challenging. A given (mouse or human) cancer may harbor tens to hundreds of genetic changes that may be identified using high throughput genomics and suitable bioinformatic approaches. An overwhelming majority of these genetic changes, however, are immunologically inactive (13). Identification of the ones that can elicit an appropriate immune response and tumor rejection is a central challenge and is addressed here.

Our studies here show that MS-defined neoepitopes can be a rich source of TRMNs. MS has been used previously for enrichment of candidate TRMNs from a pool of pre-enriched candidates identified by genomics in a mouse colon carcinoma (14) or for validation of predicted neoantigens (15). Using clinical samples of human melanoma, one of us (21) identified nearly 100,000 peptides presented by the HLA molecules; of those, 11 were shown to be neoepitopes, and of the 11, 4 elicited CD8^+^ T cell response in the patient. Here, we have combined genomic analysis to identify single-nucleotide variants (SNVs) and have directly cross-referenced the confirmed SNVs to the MS-identified peptides isolated from a mouse sarcoma; we then validated the identifications with the highly sensitive and accurate targeted MS approach with spike-in reference peptide standards. Of the 8 neoepitopes that emerged from this screening, 3 (or more than a third) were observed to be TRMNs, representing a veritable shrubbery of antigenic tumor neoepitopes. This is an extremely high yield of TRMNs, and parenthetically, is surprisingly comparable with the 4 human melanoma neoepitopes (out of 11 identified by MS) which were observed to elicit a CD8^+^ T cell response (21). None of the TRMNs identified here are known driver mutations. Consistent with previous studies, they represent passenger mutations.

Examination of the MS-identified TRMNs (Table 1 and Figure 2) and their immunological activities reveals several unexpected nuances. The most active TRMN, TGAARFDEF encoded in the *Gtf2b* gene, which elicits the most potent and complete tumor rejection in all mice immunized (Figure 2), has a moderate predicted IC_50_ (to D^d^); peptides with this level of moderate affinity for MHC I have been traditionally excluded as candidate MHC I–binding epitopes, including cancer neoepitopes. Of the other 2 active TRMNs, one (AYMKMLSSSL) has an IC_50_ of 22 nM (usually considered within the “acceptable” range), whereas the other (IGPRALDVL) with an IC_50_ of greater than 150 nM, would be excluded from consideration in traditional screening of cancer neoepitopes. Among the low-abundance TRMNs (Table 2), none have an IC_50_ value of less than 50 nM (usually considered ideal), one has an IC_50_ value of less than 100 nM, one has an IC_50_ value between 100 nM and 500 nM, and all others have IC_50_ values that range between 1,400 nM and greater than 40,000 nM. By traditional criteria then, nearly all the low-abundance TRMNs (Table 2) would be excluded as candidate TRMNs for their low affinity, and modern predictors would exclude them for their low expression level. Importantly, among the 4 neoepitopes in Table 1 with an IC_50_ of less than 100 nM, only one is a TRMN. Thus, although IC_50_ values are predictive of immunogenicity (as measured by CD8^+^ T cell responses; refs. 19, 27, 28), they are not predictors of tumor rejection and there is a great need to improve prediction of TRMNs over neoantigens with only high affinity for MHC.

The dissonance between measurable CD8^+^ T cell response and actual tumor rejection also becomes evident from the results in Figure 2. Of the 2 strong TRMNs (TGAARFDEF and AYMKMLSSSL), only TGAARFDEF elicited a measurable CD8^+^ T cell response. The neoepitope AYMKMLSSSL, which elicited complete tumor rejection in 60% of the immunized mice, did not elicit a measurable CD8^+^ T cell response. The intermediate neoepitope IGPRALDVL elicited measurable and significant CD8^+^ T cell responses. Conversely, of the 5 MHC I neoepitopes that did not elicit tumor rejection, 2 elicited a measurable CD8^+^ T cell response. Of these, the response elicited by VGPEILSSL was the strongest CD8^+^ T cell response among all neoepitopes; approximately 10% of the CD8^+^ T cells in blood in mice immunized with it were tetramer^+^. These observations clearly demonstrate that a measurable CD8^+^ T cell response is not a surrogate marker for tumor rejection.

CD8^+^ T cell responses in cancer patients are often used as surrogate markers for the activity of neoepitopes, and neoantigen-specific enriched T cells adoptively transferred into patients is emerging as a promising new treatment modality (29). In that context, analysis of CD8^+^ T cell responses to individual neoepitopes in tumor-bearing mice is revealing. No measurable CD8^+^ T cell response to TGAARFDEF (the neoepitope that elicited the strongest tumor rejection) was detected in tumor-bearing mice, even though this TRMN did elicit a robust CD8^+^ T cell response in non–tumor-bearing immunized mice (Figure 2D). The neoepitope IGPRALDVL, which elicited intermediate tumor rejection (Figure 2A) and a good CD8^+^ T cell response in naive mice (Figure 2F), elicited a significant CD8^+^ T cell response in tumor-bearing mice as well (Figure 2F). Finally, the neoepitope KYLQVASHVGL, which did not elicit tumor rejection but elicited a strong CD8^+^ T cell response in naive mice, did not elicit such response in tumor-bearing mice. Thus, measurable CD8^+^ T cell responses in tumor-bearing hosts are not predictors of anti-tumor activity in vivo and current T cell enrichment methods might miss important rejection antigens.

Discordance between CD8 responses and anti-tumor activity has been observed in many mouse (30–32) and human clinical studies (33–35). It is important to emphasize that even though we do not observe the measurable CD8 responses to be useful surrogates, the neoepitope-elicited tumor immunity is clearly CD8 dependent (Figure 2C and Figure 3C). The most likely explanation for this apparent paradox is that the CD8 response to the truly effective neoepitopes is not of a magnitude or kind that is being measured by the traditional assays used for viral and high-affinity tumor epitopes but is real and functionally effective. More sensitive and neutral assays, such as monitoring of T cell receptor amplification, may provide a solution.

MS analysis of non-abundant TRMNs shows 2 significant and potentially novel aspects. The low-abundance TRMNs (Table 2) are unable to be detected among naturally presented peptides, as generally expected because of the reported abundance bias of MS. However, pulsing of DCs in vitro with long peptides containing the mutant neoepitopes, coupled with MS analysis of peptides eluted from MHC I proteins of the DCs, allowed identification of the multiple precise neoepitopes, most of which are active in tumor rejection. Secondly, nearly all the low-abundance TRMNs investigated here have weak affinity for MHC I; with the exception of 2 peptides (which have IC_50_ values of less than 500 nM), the IC_50_ values of the low-abundance TRMNs range between 1,500 nM and greater than 40,000 nM. Peptides with such low affinities are typically not considered to bind sufficiently well and be presented by MHC I. We conclude that even though these low-abundance TRMNs have predicted low binding affinity, they were sufficiently expressed in Meth A cells, leading to immune recognition and destruction when challenged after immunization. MS may facilitate identification of the minimal epitope of such TRMNs; however, traditional predictions would not prioritize them.

Our results are based on a single model; however, they are consistent with other studies in mouse models and humans, even as the previous studies lack the sweep of our current analysis. Thus, in 2 mouse tumors, Duan et al. reported a complete lack of activity in several high-affinity neoepitopes, and instead, observed tumor rejection elicited by low-affinity neoepitopes (13). In independent human studies, Ghorani et al. and Rech et al. examined mutational and clinical outcome data from several thousand patients and concluded remarkably that the presence of high-affinity MHC-binding neoepitopes in tumors had no correlation with clinical activity (17, 18). Gross et al. had demonstrated the superior vaccination efficiency of low-affinity epitopes over high-affinity epitopes of non-mutated antigens in tumor immunotherapy (36).

In addition to peptide presentation by MHC, an immunologically relevant TRMN must be recognized by host αβ T cell receptors. Various tolerance mechanisms protect against recognition of self-peptides; therefore, active peptides must be physically distinct from their WT counterparts. Improved binding to the MHC protein through mutation, captured by the DAI score (13), can achieve this as can mutationally driven structural alterations that result in the presentation of distinct peptide surfaces. However, recent work has suggested that certain features are preferred for efficient T cell receptor recognition; mutations that increase exposed hydrophobic surface or eliminate exposed charges, for example, are expected to be more strongly correlated with immunogenicity (25, 26). In this regard, the fact that the most protective TRMN (TGAARFDEF) identified incorporated a serine-to-arginine mutation in the core of the peptide was at first puzzling. The structural modeling, however, suggested that because of large-scale structural rearrangements in response to the Ser→Arg mutation, exposed hydrophobic surface was increased and exposed charge was reduced. This result highlights the value of incorporating structural information for assessing the potency of candidate TRMNs.

Altogether, our studies reveal a number of nuances to our understanding of antigen presentation, including presentation of cancer neoepitopes and TRMNs. These studies also yield precious insights on prioritization of targets for personalized neoepitope-based cancer vaccines and cancer immunotherapy.

## Methods

### Cells.

Meth A cells (1–10 × 10^8^) and BMDCs (5 × 10^7^ cells) were stored as dry cell pellets at −80°C until use.

### Exome and RNA sequencing and bioinformatics analysis.

The exome and transcriptome of the Meth A tumor cell line were sequenced using both Illumina and ION Torrent sequencing platforms and putative SNVs were predicted using the CCCP tool from the GeNeo toolbox for genomics-guided neoepitope prediction available on the public Galaxy server maintained at https://neo.engr.uconn.edu GeNeo is an expanded version of the Epi-Seq pipeline previously published by our group (13). The CCCP tool calls SNVs from both sequencing technologies using 2 somatic variant callers, Strelka and the somatic variant caller version of SNVQ (37), and generates consensus calls along with exome/RNA coverage information for each SNV position. The EpitopeFinder tool of GeNeo was then used to predict MHC I binding affinities and DAI scores (13) for SNVs called by CCCP. The list of long mutated peptides created by EpitopeFinder was used for MSMS peptide identification. Gene expression estimation from RNA-seq data was performed by using IsoEM2 algorithm (38). Gene expression was reported as transcripts per million (TPM) units.

### Generation of antibody-crosslinked beads.

Anti–MHC I monoclonal antibody was purified from the supernatant of HIB 34.1.2 (gift from Angel Miguel Garcia-Lora, Hospital Universitario Virgen de las Nieves, Granada, Spain) CELLLine CL-1000 flasks (Sigma-Aldrich) using Protein A–Sepharose 4B beads (Invitrogen). Antibodies were cross-linked to Protein A–Sepharose 4B beads at a concentration of 5 mg of antibodies per 1 mL volume of beads. For this purpose, the antibodies were incubated with the Protein A–Sepharose 4B beads for 1 hour at room temperature. Chemical cross-linking was performed by the addition of dimethyl pimelimidate dihydrochloride (Sigma-Aldrich) in 0.2 M sodium borate buffer pH 9 (Sigma-Aldrich) at a final concentration of 20 mM for 30 minutes. The reaction was quenched by incubation with 0.2 M ethanolamine pH 8 (Sigma-Aldrich) for 2 hours. Cross-linked antibodies were kept at 4°C until use.

### Purification of MHC I–eluted peptides.

MethA cells or APCs were lysed in phosphate buffered saline containing 0.50% sodium deoxycholate (Sigma-Aldrich), 0.2 mM iodoacetamide (Sigma-Aldrich), 1 mM EDTA, 1:200 Protease Inhibitor Cocktail (Sigma-Aldrich), 1 mM phenylmethylsulfonylfluoride (Roche), and 1% octyl-β-D glucopyranoside (Sigma-Alrich) at 4°C for 1 hour. The lysis buffer was added to the cells at a concentration of 10^8^ cells/mL. Lysates were cleared by centrifugation with a table-top centrifuge (Eppendorf Centrifuge) at 4°C at 20,000 *g* for 50 minutes. MHC I molecules were purified by incubating the cleared lysates with HIB antibodies cross-linked to Protein A–Sepharose 4B beads in affinity columns for 3 hours at 4°C. A ratio of 100 μL of cross-linked beads per 10^8^ cells was applied. The affinity columns were then washed as follows: 2 column volumes of 150 mM sodium chloride (NaCl; Carlo-Erba) in 20 mM Tris-HCl pH 8, 2 column volumes of 400 mM NaCl in 20 mM Tris-HCl pH 8, and again 2 column volumes of 150 mM sodium chloride in 20 mM Tris-HCl pH 8. Finally, the beads were washed in 1 column volume of 20 mM Tris-HCl pH 8. MHC complexes and the bound peptides were eluted at room temperature by adding twice a volume of 1% trifluoroacetic acid (TFA) equivalent to or slightly higher than the volume of beads present in the column. Sep-Pak tC_18_ 96-well plates (Waters), preconditioned with 1 mL of 80% acetonitrile (ACN) in 0.1% TFA and then with 2 mL of 0.1% TFA, were used for the purification and concentration of MHC I peptides. Elutions containing MHC I molecules were loaded in the Sep-Pak tC_18_ 96-well plates and the C_18_ wells were then washed with 2 mL of 0.1% TFA. The MHC I peptides were eluted with 500 μL of 28% ACN in 0.1% TFA. MHC I peptides containing elutions were transferred into Eppendorf tubes. Recovered peptides were dried using vacuum centrifugation (Thermo Fisher Scientific) and stored at −20°C.

### LC-MS/MS analyses for the discovery of neoepitopes.

Prior to MS analysis, MHC I peptide samples were resuspended in 12 μL of 2% CH_3_CN and 0.1% formic acid (FA). Then, 3 μL of sample was loaded on the column for each measurement by LC-MS/MS.

The LC-MS system consists of an Easy-nLC 1200 (Thermo Fisher Scientific) coupled on-line to Q Exactive HF and or HF-X mass spectrometer (Thermo Fisher Scientific). Peptides were separated on a 450-mm homemade column of 75-μm inner diameter packed with ReproSil Pur C_18_–AQ 1.9-μm resin (Dr. Maisch GmbH). The analytical separation was performed for a period of 125 minutes using a gradient of H_2_O/FA 99.9%/0.1% (solvent A) and CH_3_CN/FA 80%/0.1% (solvent B). The gradient was run as follows: 0 minutes 2% B, then to 5% B at 5 minutes, 35% B at 85 minutes, 60% B at 100 minutes, 95% B at 105 minutes, 95% B at 110 minutes, 2% B at 115 minutes, and 2% B at 125 minutes at a flow rate of 250 nL/minute.

The mass spectrometer was operated as follows for the discovery of neoepitopes. Full-scan MS spectra were acquired in the Orbitrap from *m*/*z* = 300–1,650 at a resolution of 60,000 (*m*/*z* = 200) with a maximum injection time of 20 ms. The auto gain control (AGC) target value was set to 3 × 10^6^ ions. MS/MS spectra were acquired at a resolution of 15,000 (*m*/*z* = 200) using the data-dependent acquisition (DDA) method on the 10 most abundant precursor ions (if present). Each precursor ion was sequentially isolated with an isolation window of 1.2 *m*/*z*, activated by higher-energy collision dissociation (HCD) with a normalized collision-energy (NCE) of 27. Ions were accumulated to an AGC target value of 1 × 10^5^ with a maximum injection time of 120 ms. In the case of assigned precursor ion charge-state of 1, and from 6 and above, no fragmentation was performed. Selected ions were dynamically excluded for additional fragmentation for 20 seconds and the peptide match option was disabled. A few DDA analyses were also performed with an inclusion list of selected peptides.

### Identification of peptides by MS.

We employed the MaxQuant platform (39) version 1.5.5.1 to search the peak lists against a FASTA file containing the mouse proteome (Mus musculus_UP000000589_10090, the reviewed part of UniProt, with no isoforms, including 24,907 entries downloaded in June 2016) concatenated to a list of 3,783 long peptides (up to 31 aa) encompassing the nonsynonymous somatic mutations described previously. Peptides with a length between 8 and 25 aa were allowed. The second peptide identification option in Andromeda was enabled. The enzyme specificity was set as unspecific. An FDR of 5% was required for peptides and no protein FDR was set. The initial allowed mass deviation of the precursor ion was set to 6 ppm and the maximum fragment mass deviation was set to 20 ppm. Methionine oxidation and N-terminal acetylation were set as variable modifications. The peptide output files summarizing MaxQuant result files are provided as Supplemental Table 1. The values of peptides intensities were log2 transformed and normalized. Briefly, for each sample the first, second, and third quartiles (q1, q2, q3) were calculated from the distribution of all values. The median (q2) was subtracted from each value to center the distribution. Then we divided by the width in an asymmetric way. All values that were positive after subtraction of the median were divided by q3 – q2, while all negative values were divided by q2 – q1. Average normalized intensity was calculated for each MS-detected neoepitope.

### Validation of neoepitopes with PRM.

Synthetic peptides labeled with heavy isotopes were purchased as crude (PEPotec Immuno Custom peptide libraries grade 3) from Thermo Fisher Scientific (Paisley, PA49RE) with an isotopic purity greater than 99%. The labeled amino acid in each peptide is indicated in Supplemental Figures 2 and 3. The peptides were mixed, ensuring that each peptide was spiked into the MethA peptidomic samples with a concentration of 1 pmol/μL. For quality control, before spiking the peptides, we monitored the presence of residual interferences of “light” peptides by measuring separately each peptide with the method described in the following information. The same peptide mixture was spiked into the peptidomic sample eluted from the APCs as a negative control. We measured the spiked-in samples first with the DDA method described previously and then with the PRM method. For PRM acquisition, the mass spectrometer was operated at a resolution of 60,000 (at *m*/*z* = 200) for full scan MS, scanning a mass range from 300–1,300 *m*/*z* with a maximum ion injection time of 45 ms and an AGC target value of 3 × 10^6^. Then each peptide was isolated with an isolation window of 2.0 *m*/*z* prior to ion activation by HCD (NCE = 27). Targeted MS/MS spectra were acquired at a resolution of 30,000 (at *m*/*z* = 200) with a maximum ion injection time of 60 ms and an AGC target value of 2 × 10^5^. The data were then processed and analyzed by Skyline (MacCoss Lab Software) and an ion mass tolerance of 0.02 *m*/*z* was used to extract fragment ion chromatogram. Raw data were converted into Mascot generic format by MSConvert (Proteowizard) to extract matched peak lists for heavy peptide and light counterpart for visualization of the MSMS spectra. The assessment of MS/MS matching was done by pLabel (Version 2.4.0.8, pFind Studio; http://pfind.ict.ac.cn/software/pLabel/index.html) and Skyline.

### Mice and tumors.

BALB/cJ mice (6-week-old females) were purchased from the Jackson Laboratory and maintained in our specific pathogen–free mouse facilities under approval from the Institutional Animal Care and Use Committee. Meth A cells that have been in our laboratory since 1988 were originally obtained from Lloyd J. Old. Meth A ascites cells were used for passage. All the tested neoepitopes of the BALB/cJ Meth A fibrosarcoma were synthesized by JPT Peptide Technologies GmbH and were greater than 95% pure.

### BMDCs.

Bone marrow cells (2–3 million per 10 cm^2^ bacteriological Petri dishes) of 6- to 8-week-old mice were cultured in complete RPMI supplemented with 20 ng/mL recombinant murine granulocyte-macrophage colony-stimulating factor (GM-CSF) (Peprotech) and incubated at 37°C for 7 days to generate GM-CSF BMDCs.

### Immunization and tumor rejection.

Day 7 BMDCs were pulsed with 40 μg of the neoepitope (1 μL of a 20 mM neoepitope solution was added to 7.5 million BMDCs in 200 μL RPMI medium for final peptide concentration of 100 μM). The pulsed BMDCs were washed 4 times and used to immunize a single mouse. Mice were randomized before being used for immunization with individual peptides to control for any cage effects. All immunizations were performed in the presence of CTLA4 blockade, using the IgG2b isotype (clone 9D9, Bio X Cell), administered with the second immunization and every 3 days after tumor challenge except for peptide TGAARFDEF. Tumor diameters were measured in 2 dimensions and the average diameter determined.

### TCI.

TCI quantitates and integrates all 3 aspects of tumor growth: inhibition, period of tumor size stability, and tumor shrinkage (complete response), as published previously (23).

### CD8 depletion in vivo.

BALB/cJ mice were injected with 250 μg of isotype control (*InVivo*MAb rat IgG2b isotype control, Bio X Cell) or CD8-depleting antibody (*InVivo*MAb anti–mouse CD8α, rat IgG2b, clone 2.43, Bio X Cell) antibodies 6 days before the first immunization (priming phase). The CD8^+^ cells remained completely depleted in all mice until day 6; after that the recovery was variable.

### Flow cytometry.

The antibody specific for Fixable Viability Dye eFluor 780 was purchased from eBioscience. PE tetramer specific for each neoepitope was synthesized, validated, and tested by MBL International Corporation. Tetramer testing was performed prior to product release according to standard operating procedures of MBL, and the tetramers were further tested for specificity in our laboratory. The FITC-labeled antibody specific for CD8 (clone KT15) was purchased from the same company. BV510 CD3 (clone 17A2) was purchased from Biolegend. Mouse FcR blocking reagent was purchased from Miltenyi Biotec. Flow cytometry was performed using Miltenyi Biotec MACSQuant analyzer. The analysis was done using FlowJo software (FlowJo LLC).

### Structural modeling of WT/neoepitope peptide-MHC pairs.

Structural modeling of peptide/MHC complexes used Rosetta (40) molecular modeling software via the PyRosetta 4.0 interface (41) and the ref2015 energy function (42). Template structures for modeling were chosen based on presence of matching allele, peptide length, P2 anchor, and PΩ anchor, in order of preference. The template for KYLQVASHV was Protein Data Bank (PDB) ID 4Z76; for TGAARFDEF it was 5KD7 (43, 44). For both cases, coordinates from the first molecule in the asymmetric units were used and all atoms from subsequent molecules were removed. Prior to modeling new peptides, the template peptide-MHC structures were energy minimized for 5 cycles of the Rosetta FastRelax protocol (45), which alternates between energy minimization and side-chain repacking to bring structures to local energy minima.

After minimization, the desired peptide sequence was computationally introduced using the PyRosetta mutate_residue function to replace sequential residues in the peptide with the desired amino acid in the target peptide, maintaining peptide backbone atom coordinates and using idealized side-chain atom coordinates. This was followed by kinematic loop remodeling of the peptide backbone via multiple cycles of random perturbation of the φ/ψ dihedral angles, then repacking amino acid side chains with the LoopMover_Perturb_KIC protocol (46) applied to a simplified centroid representation of the peptide. A final high-resolution refinement of all peptide atoms via the LoopMover_Refine_KIC protocol was then performed. Because of the extensive conformational diversity allowed by stochastic modeling, we generated 10,000 decoys for each peptide/MHC complex to sufficiently sample the available phase space. In each case, the lowest energy decoy was selected as the final model. RMS deviation of atomic positions of peptide common or backbone heavy atoms between WT and mutant peptides was calculated, and models were inspected visually for differences in structural features using Discovery Studio. As expected from the stochastic modeling, the final models exhibited deviations in the peptide backbone from their respective template structures; in terms of RMS deviations these were 0.47 Å for KYRQVASHV, 0.66 Å for KYLQVASHV, 1.42 Å for TGAASFDEF, and 0.76 Å for TGAARFDEF.

### Statistics.

*P* values were calculated using a 2-tailed *t* test or 1-way ANOVA test by GraphPad Prism 5.0 (GraphPad). Dunnett’s or Tukey’s tests were performed as follow-up tests to ANOVA to correct for multiple comparisons for TCI and AUC comparisons, respectively. The adjusted *P* value for each comparison is reported. *P* < 0.05 was considered statistically significant.

### Study approval.

All animal studies were carried out upon under approval from the Institutional Animal Care and Use Committee of the University of Connecticut School of Medicine.

## Author contributions

PKS, HEN, MBS, GC, and BMB conceptualized and designed the research, analyzed the data, and wrote the manuscript. HEN, JM, WLC, and HSP did the experiments. IIM gave the guidance for analysis of sequencing data, and TS analyzed the sequencing data. GLJK performed the modeling of peptide/MHC I complexes.

## Supplementary Material

Supplemental data

## Figures and Tables

**Table 2:**
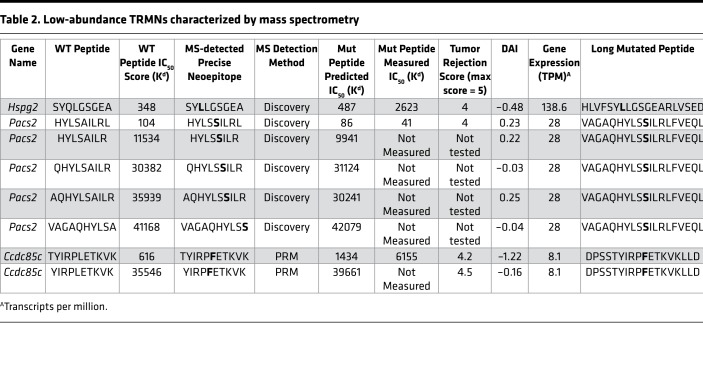
Low-abundance TRMNs characterized by mass spectrometry

**Table 1:**
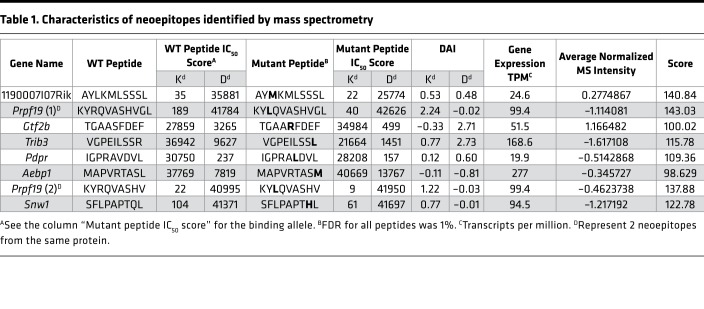
Characteristics of neoepitopes identified by mass spectrometry

## Version 1 06/20/2019

In-Press Preview

## Version 2 07/25/2019

Electronic publication
