# Supplementary material for: Mass spectrometry–driven exploration reveals nuances of neoepitope-driven tumor rejection
Source: JCI Insight. 2019 Jul 25;4(14):e129152. doi: 10.1172/jci.insight.129152 (PMC6675551; doi:10.1172/jci.insight.129152)
Supplement: Supplemental data [file jciinsight-4-129152-s175.pdf]

Summary of Supplemental material

Supplemental Table 1:
List of all MHC-I peptides identified by discovery MS immunopeptidomics from MethA cells

Supplemental Table 2:
List of MHC-I peptides identified by discovery MS immunopeptidomics from the three long peptides loaded on BMDCs

Supplemental Figure 1:
Characteristics of MHC-I ligands identified by MS in MethA cells, including peptide length distribution (A), the consensus binding motifs of MHC-H2 Kd, Dd, and Ld allotypes as revealed by clustering the 9 mer peptides (B), and the averaged normalized MS intensity of MHC-I peptides including neoepitopes identified in several biological replicates.

Supplemental Figure 2:
Targeted MS-based validation of the MS identified MHC-I neoepitopes KYLQVASHV (A), KYLQVASHVGL (B), VGPEILSSL (C), IGPRALDVL (D), SFLPAPTHL (E), MAPVRTASM (F) and MAPVRTASM with methionine oxidation (G).

Supplemental Figure 3:
Targeted MS-based identification of two MHC-I neoantigens TYIRPFETKVK (A) and YIRPFETKVK (B) from Ccdc85c gene in BMDCs loaded with the long peptide DPSSTYIRPFETKVKLLD.

Supplemental Figures:

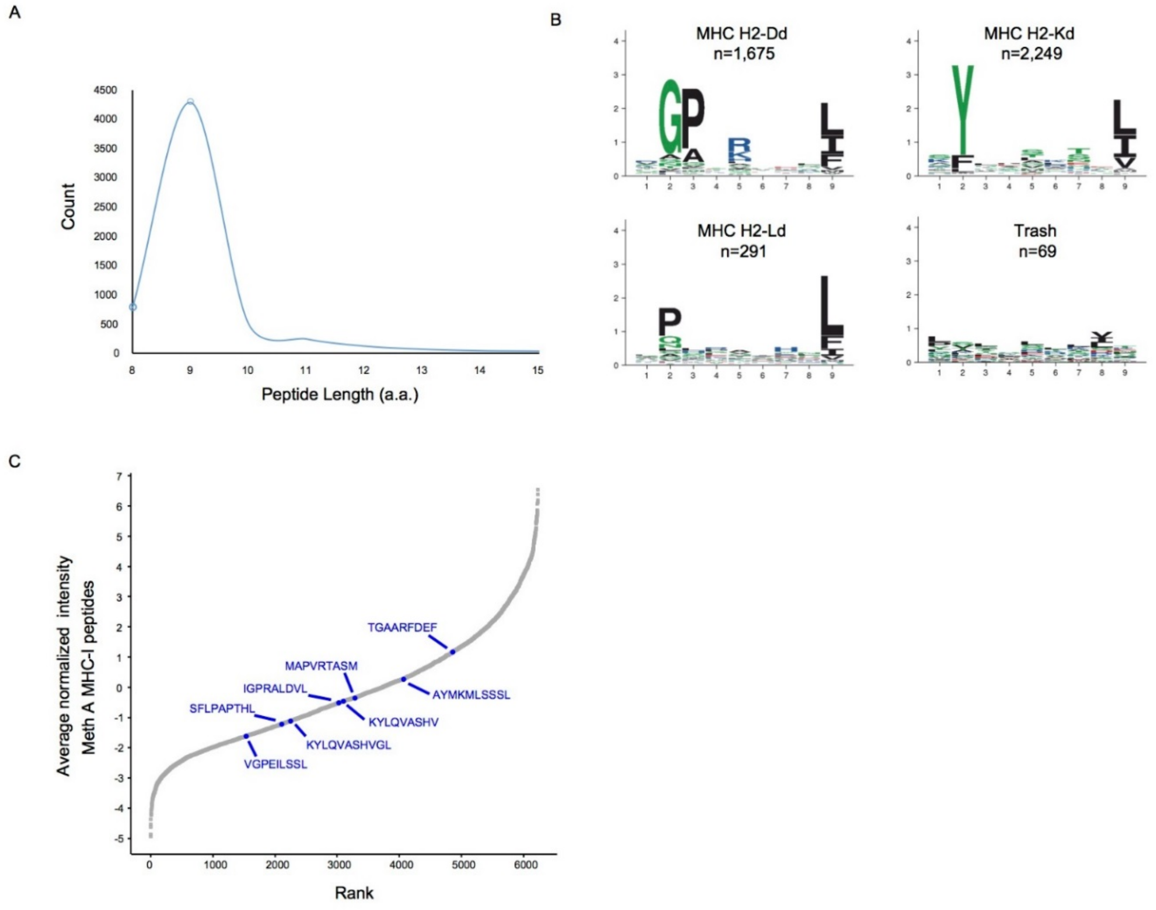

Supplemental figure 1: A) Length distribution of 6,209 MHC-I peptides identified in Meth A with immunopeptidomics. B) Clustering of 4,284 MHC-I 9 mer peptides reveals the consensus binding motifs of MHC-H2 Kd, Dd, and Ld allotypes. C) Averaged normalized MS intensity of MHC-I peptides identified in several biological replicates. MS identified neoepitopes are highlighted.

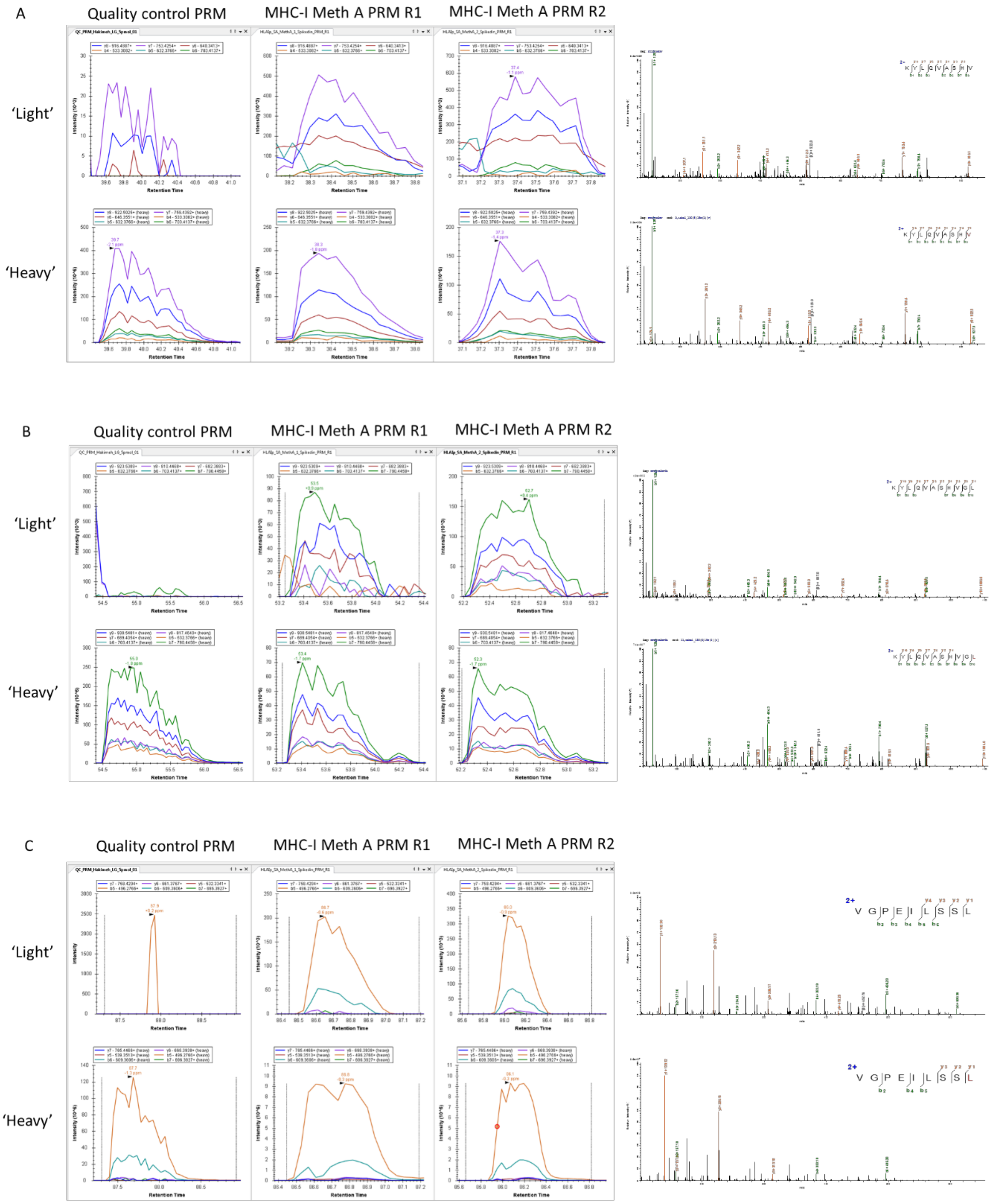

Supplementary Figure 2 (continued on next page).

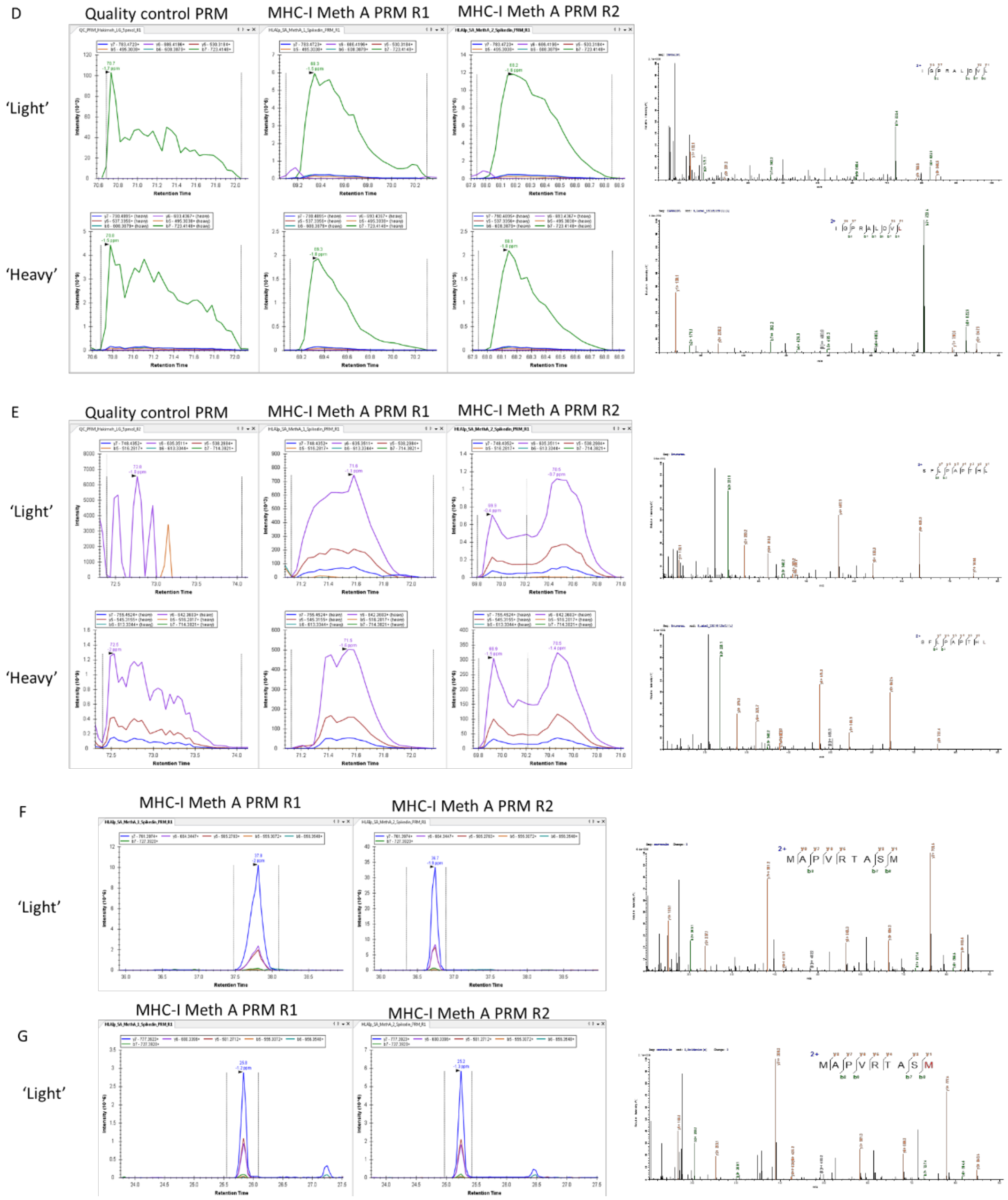

Supplemental Figure 2: Validation based on targeted MS analysis of the additional six MS identified MHC-I neoepitopes (A-G). Matched peak lists for the 'heavy' and 'light' ions were extracted and monitored. First, the absence of 'light' peptide and the presence of the 'heavy' peptide were confirmed by PRM as a quality control measure in the synthetic peptide samples (upper left and lower left, respectively). Then, the co-elution of the synthetic 'heavy' and endogenous 'light' fragment ions was measured by PRM in two independent Meth A MHC-I immunopeptidomics samples. Representative resulting MS/MS spectra of the 'light' and 'heavy' counterparts are provided. The peptide MAPVRTASM was exceptionally detected by PRM without a spike-in of a 'heavy' counterpart, in the native form (F) and with a methionine oxidation (G).

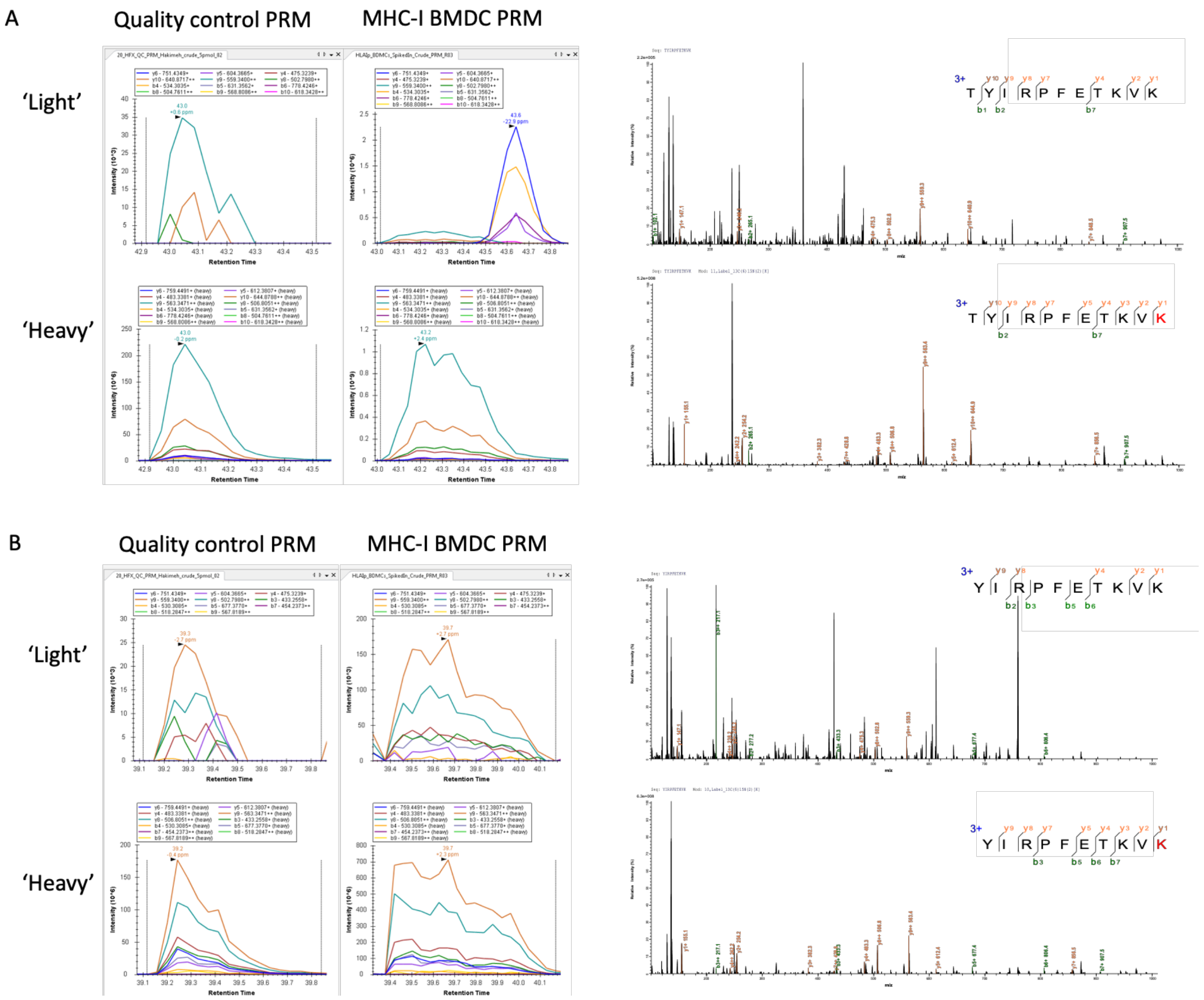

Supplemental Figure 3: Targeted PRM MS analysis of two MHC-I neoantigens TYIRPFETKVK (A) and YIRPFETKVK (B) from Ccdc85c gene identified in BMDCs loaded with the long peptide DPSSTYIRPFETKVKLLD. Matched peak lists for the 'heavy' and 'light' ions were extracted and monitored. First, the absence of 'light' peptide and the presence of the 'heavy' peptide were confirmed by PRM as a quality control measure in the synthetic peptide samples (upper left and lower left, respectively). Then, the co-elution of the synthetic 'heavy' and endogenous 'light' fragment ions was measured by PRM. Representative resulting MS/MS spectra of the 'light' and 'heavy' counterparts are provided.
